# Integrating Mental Health and Psychosocial Support Into Health Facilities in Conflict Settings: A Retrospective Review From Six African Countries

**DOI:** 10.3389/fpubh.2020.591369

**Published:** 2020-12-11

**Authors:** Ida Andersen, Rodolfo Rossi, Mamie Nouria Meniko Yabutu, Ives Hubloue

**Affiliations:** ^1^Health Unit, International Committee of the Red Cross, Geneva, Switzerland; ^2^Research Group on Emergency and Disaster Medicine, Vrije Universiteit Brussel, Brussels, Belgium

**Keywords:** mental health and psychosocial support (MHPSS), sexual violence, Africa, primary healthcare (PHC), International Committee of the Red Cross (ICRC), armed conflict, lay counselors

## Abstract

**Introduction:** The International Committee of the Red Cross runs an increasing number of mental health and psychosocial programmes integrated into health facilities in conflict settings across Africa. This study looks at changes in symptoms of psychological distress and impaired functioning among patients supported through such programmes.

**Material and Methods:** Between January and December 2019, 5,527 victims of violence received mental health and psychosocial support in 29 health facilities in Burundi, Central African Republic, Democratic Republic of the Congo, Mali, Nigeria and South Sudan. Symptoms of psychological distress (IES-R or DASS21) and daily functioning (ICRC scale) were assessed before and after the intervention. Logistical regression models were used to measure associations between these symptoms and the other variables.

**Results:** Factors associated with high distress prior to receiving support included age (peaking at 45–54 years), intervening within three months, rape, caretaker neglect, internal displacement, secondary education level and referral pathway. Anxiety levels in particular were higher among victims of violence committed by unknown civilians, the military or armed groups. Low functioning was associated with divorce, grief and violence committed by the military or armed groups. Following the intervention, the vast majority of patients reported reduced psychological distress (97.25% for IES-R and 99.11% for DASS21) and improved daily functioning (93.58%). A linear trend was found between number of individual sessions and reduction in symptoms of distress. Financial losses were associated with less reduction in symptoms of depression and stress.

**Discussion:** To further address the mental health and psychosocial needs of victims of violence, intervening quickly and increasing the number of individual sessions per patient is crucial. This requires proximity—being in the right place at the right time—which is challenging when working in stable health structures. Symptoms of depression should not be overlooked, and financial losses must be addressed in order to holistically meet the needs of victims of violence.

## Introduction

Mental health and psychosocial support (MHPSS) is increasingly recognized as an integral part of humanitarian assistance offered to conflict-affected populations. MHPSS in conflict settings is also a rapidly growing area of research as scholars and practitioners seek to identify predictors of psychological distress and evidence-based approaches to treatment.

With regards to predictors of distress, a systematic review identified *age* as an important factor for prevalence of post-traumatic stress disorder (PTSD), with a sharp incline in childhood years—peaking at around 25 years—and a decline after 55 years of age (1). A cross-sectional study of patients in a hospital run by Doctors Without Borders (MSF) in Central African Republic (CAR), found high prevalence rates of PTSD (33%), acute stress (17%), insomnia (63%), anxiety (45%), and depression (41%), and identified *rape, female gender* and *high anxiety* and *depression* as the main predictors of stress (2). A study of East-African conflict survivors found that stigmatization was associated with risk of PTSD and diminished likelihood of spontaneous remission (3).

In terms of efficacy of MHPSS interventions in humanitarian settings, a Cochrane study found substantial evidence for reduction in symptoms of Posttraumatic Stress Disorder (PTSD) and depression in adults, while moderate evidence was found for reduction of anxiety in adults (4). Also, a recent umbrella review found a relatively large amount of evidence pointing to the benefit of psychosocial interventions on various mental health outcomes in low and middle-income countries, at the same time pointing to the need for more research to enhance the evidence base (5).

The MHPSS programs run by the International Committee of the Red Cross (ICRC) target a range of populations affected by armed conflict and other situations of violence (6). Programs are located in areas where the civilian population can safely access services addressing MHPSS needs deriving from ongoing fighting or political tension.

The main type of program targets victims of violence, including sexual violence, and is implemented either in health facilities in collaboration with the Ministry of Health or at community level through, for example, the National Red Cross/Red Crescent Society. Broadly speaking, activities fall within three categories: capacity-building carried out mainly by the ICRC team of expatriate and resident psychologists, awareness-raising and direct service provision carried out most often by local partners trained and supervised by ICRC MHPSS teams.

Compared to MHPSS programs at community level, integrating local health-care facilities presents certain advantages in terms of *proximity to medical care, discretion* and greater potential for *local ownership and sustainability* insofar as efforts are made to ensure gradual recognition by the Ministry of Health of the role of the counselor in the concerned health facilities. On the other hand, disadvantages may include *less influence* on the selection and working conditions of the counselors and on the types of patients referred to her/him and *less proximity* to direct victims of armed conflict as health facilities are permanent structures whereas community-level activities offer more flexibility in terms of following the movements of armed conflict. In recent years, standardized monitoring has become an integral part of ICRC MHPSS programs in conflict-affected areas around the world. This is the first review of ICRC MHPSS programmes in health facilities across Africa.

## Materials and Methods

### Study Design

This is a non-controlled review of 5,527 victims of violence who received MHPSS in 29 health facilities across Burundi, the CAR, the DRC, Mali, Nigeria and South Sudan between January and December 2019.

### Participant Selection

In 2019, the ICRC supported 248 primary healthcare facilities (PHC) and 123 hospitals in conflict settings across Africa. Among these, ICRC-supported MHPSS services were available in 25 PHCs and four hospitals, namely: eight PHCs in Burundi, one hospital and three PHCs in CAR, eight PHCs in DRC, two hospitals and four PHCs in Mali, one PHC in Nigeria as well as one hospital and one PHC in South Sudan. The services were offered to both adults and children seeking support (i.e., there were no exclusion criteria) and all available MHPSS data regarding the patients were included in this study.

### The MHPSS Intervention

The ICRC MHPSS victims of violence programmes in health-care facilities are carried out in three phases:

#### Pre-assessment

At the time of enrolment, a local counselor working inside an ICRC-supported health facility assesses levels of psychological distress and functioning using standardized psychometric tools (see section Sources of Data). These monitoring tools have been chosen by our senior MHPSS specialists on the basis of literature reviews and practical field experience as the best fit for the target beneficiaries. Given that most first-line counselors have little formal training in clinical psychology, scales based on *self-reporting* by patients as opposed to clinical judgement were an important requirement for the selection.

#### Individual Sessions

Following the pre-assessment, a psychological treatment plan is defined based on the most pressing needs and symptoms, and individual sessions are offered on a weekly basis. Although variations exist from context to context, counselors generally follow a six-step methodology (7) consisting of:

1. Identifying the most pressing problem
2. Brainstorming for solutions to the most pressing problem
3. Exploring pros and cons of each possible solution
4. Choosing the most promising solution given the available resources
5. Planning the implementation of the solution by the patient
6. Evaluation of the implementation and repetition of steps as needed.

The ICRC MHPSS team in the country provides regular training sessions and case supervision, along with referrals to local service providers according to needs and availability.

#### Post-assessment

At the end of the treatment, a closing session takes place during which levels of psychological distress and functioning are reassessed using the same tools as the pre-assessment.

### Sources of Data

The data used in this study comes from the ICRC MHPSS Excel database “Pearl” containing patient demographics (ten variables), trauma history (nine variables), the type of support received (five variables) as well as pre- and post-assessment scores on psychological distress and daily functioning. The Pearl was developed by senior ICRC MHPSS psychologists and psychiatrists and the variables and scales were selected to give field counselors the necessary information to develop a tailor-made treatment plan for each patient.

#### Psychological Distress

There are currently two self-reported measures of psychological distress which the MHPSS field teams are free to choose from: The Impact of Events Scale Revised (IES-R) and the Depression, Anxiety and Stress Scale with 21 items (DASS21). Both the IES-R and the DASS21 are suitable for use across different cultural settings (8–11). While the former measures symptoms of PTSD and is particularly relevant to patients who have *recently* been exposed to a *particular* violent event, the latter may be more adapted to patients who have undergone *multiple* experiences of violence and/or present more *long-term* and chronic reactions. In 2019, for MHPSS programs in health facilities in Africa, the IES-R was used in Burundi, CAR, Mali and Nigeria (3,013 patients), whereas the DASS21 was used in the DRC and South Sudan (2,514 patients).

The IES-R scale contains an Intrusion (eight items), an Avoidance (eight items) and a Hyperarousal (six items) subscales that are rated from zero to four and generate total scores ranging from zero to 88. Four severity categories of the total score have been proposed (12), with 39 being the cut-off score for the highest-severity category. As the pre-scores of many ICRC MHPSS patients are higher than 39, a fifth IES-R severity category was added in this study. It categorizes IES-R total scores ranging from 64 to 88 as “extremely severe” (Table 1). The DASS21 contains three subscales for Depression, Anxiety and Stress. Each subscale contains seven items, which can be scored from zero to two, leading to a total score ranging from zero to 42. Formal cut-off scores have been defined (13) for categorizing scores on each subscale as normal, mild, moderate, severe or extremely severe.

**Table 1:** Categorization of DASS21, IES-R and functioning scores used in this study.

	**IES-R total**	**DASS21 Depression**	**DASS21 Anxiety**	**DASS 21 Stress**	**ICRC Functioning**
Normal	0–23	0–9	0–7	0–14	0–2
Mild	24–32	10–13	8–9	15–18	3–5
Moderate	33–38	14–20	10–14	19–25	6–8
Severe	39–63	21–27	15–19	26–33	9–11
Extremely severe	64–88	29+	20+	34+	12–14

#### Functioning

The ICRC has developed an Africa-specific functionality scale using the free listing method (14) (Table 2). The scale differentiates between women, men and children and contains seven items scored as 0 (not capable), 1 (capable, but with more difficulty than before the violent event) or 2 (capable). Total scores range from zero to 14 and were categorized in this study as normal, mild, moderate, severe or extremely severe. In 2019, for MHPSS programs in health facilities in Africa, the ICRC functionality scale was used in Burundi, the CAR, the DRC and Mali. In Nigeria and South Sudan levels of functionality were unfortunately not recorded.

**Table 2:** ICRC functionality scale for Africa.

**ICRC Functionality Scale for Africa– 14 items**
The *woman* is:
Able to work (go to the field, to the market…)
Able to take care of her children
Able to take care of her house
Able to take care of herself (hygiene…)
Able to sleep
Able to interact with others (relationship with others, intimacy…)
Able to take part in social activities (church/mosque, ceremonies, women's group, choir…)
The *man* is:
Able to work
Able to sleep
Able to interact with others (relations with others, sexual relations…)
Able to take part in social activities (church/mosque, ceremonies, friends…)
Able to provide the needs of the family
Able to take care of himself (hygiene…)
Able to leave the house
The *child* is
Able to speak
Able to play
Able to walk
Able to interact with others
Able to eat
Able to sleep
Able to go and to work at school

### Data Management and Statistical Analysis

All categorical data were numerically coded. Quantitative/continuous variables (i.e., pre- and post- scores) were either kept as such or categorized depending on the type of analysis. Categorization of continuous variables was done either by identifying the median to divide the study participants in two even-sized groups or by using established clinical cut-offs (see section Sources of Data).

The dataset was created in Microsoft Excel with two independent data clerks to control for potential typing mistakes. The electronic dataset was protected by a password, which was changed every three months. The dataset was transferred to STATA™, version MP 16.0 for analysis.

All quantitative variables were explored by defining their means (and standard deviation), medians and quartiles. Comparisons of means were tested through the t-test, and the corresponding *p*-value was reported; 95% confidence intervals (95% CI) were calculated around means and means differences. Categorical variables were explored through percentages and tested using the Chi^2^ test to retrieve the corresponding *p*-value; 95% CIs were calculated around these percentages.

To measure associations between variables (crude and multivariable), logistic regression models were fitted to calculate odds ratios (OR) with corresponding 95% CIs and *p*-values from the Wald test. All variables were initially explored in a crude model and their results were only presented if they were found statistically significant.

## Results

### The Study Population

The study population (Table 3) included 5,527 victims of violence in Burundi (40%), the DRC (40%) the CAR (11%), South Sudan (6%), Mali (2%), and Nigeria (1%). The vast majority were female (81%) and residents (79%) as opposed to displaced. There was a diversity of age, educational background, current occupation, civil status and number of children. Around one-third of the patients consulted an MHPSS practitioner within 48 h following exposure to violence, one-third between two and 90 days and one-third beyond 90 days.

**Table 3:** Characteristics of the study population.

	***n***	**%**
**Country (*****N*** **=** **5,527)**		
Burundi	2,201	39.82
Central African Republic	609	11.02
Democratic Republic of the Congo	2,194	39.7
Mali	121	2.19
Nigeria	82	1.48
South Sudan	320	5.79
**Gender (*****N*** **=** **5,512)**		
Male	1,034	18.76
Female	4,478	81.24
**Age (*****N*** **=** **3,785)**		
2–17	434	11.47
18–24	823	21.74
25–34	1,350	35.67
35–44	705	18.63
45–81	473	12.5
**Education level (*****N*** **=** **5,427)**		
Illiterate	1,624	29.92
Basic	2,437	44.91
Medium	1,281	23.6
High	84	1.57
**Current occupation (*****N*** **=** **5,004)**		
Unemployed	1,410	28.18
Student	559	11.17
Farming	1,902	38.01
Other job	1,133	22.64
**Civil status (*****N*** **=** **5.407)**		
Single (incl. children)	1,433	26.5
Married	2,844	52.6
Partner abroad	18	0.33
Partner missing	12	0.22
Divorced/Separated	646	11.95
Widow/er	447	8.27
Other	7	0.13
**Number of children (*****N*** **=** **4,094)**		
0	540	13.19
1	494	12.07
2	547	13.36
3	599	14.63
4	557	13.61
5	421	10.28
6	377	9.21
7–20	559	13.65
**Status (*****N*** **=** **5,474)**		
Resident	4,298	78.52
Migrant	1,165	21.28
Other	11	0.2
**Main types of violence highlighted by the patient during the first consultation**		
Physical violence (*N* = 5.139)	2,672	52.01
Witness to physical violence (*N* = 4.972)	766	15.41
Rape (*N* = 5.122)	1,156	22.57
Attempted rape (*N* = 4,934)	101	2.05
Incest (*N* = 4.911)	18	0.37
Forced marriage (*N* = 4.911)	47	0.96
Forced prostitution(*N* = 4.909)	15	0.31
Trafficking/Smuggling (*N* = 4.908)	13	0.26
Kidnapping incl. sexual violence (*N* = 4.908)	79	1.61
Kidnapping excl. sexual violence (*N* = 4.913)	27	0.55
Killing of family member/Loved one (*N* = 4.930)	384	7.79
Disappearance of family member (*N* = 4.908)	243	4.95
Forced recruitment by armed group (*N* = 4.908)	26	0.53
Torture (*N* = 4.904)	327	6.62
Insults/Threats (*N* = 5.041)	697	11.83
**Other factors of vulnerability highlighted by the patient during the first session (*****N*** **=** **5.527)**		
Destroyed/Lost property and/or income	1,590	28.77
Mother head of household	501	9.06
Natural death of loved one <2 years ago	552	9.99
Natural death of loved one more than 2 years ago	481	8.7
Missing a relative	140	2.53
Caretaker neglect (for minors only)	255	4.61
Severe or chronic medical/Physical condition	328	5.93
Severe or chronic mental health condition	79	1.43
Highly stigmatized disease(s)	97	1.76
Congenital abnormality	32	0.58
Experience of discrimination/Stigma/Marginalization	400	7.24
Lack of social support/Network	1,175	21.26
Past incarceration without solitary confinement	28	0.51
Past incarceration with solitary confinement	26	0.47
Forced to flee	591	10.69
Accidents	223	4.03
Other	295	5.34
**Place of violence (*****N*** **=** **4.767)**		
Home	2,847	59.72
School/Work	356	7.47
On the road/While going somewhere	682	14.31
During combat	592	12.42
While fleeing violence/On the move	144	3.02
IDP/Refugee camp	12	0.25
Other	133	2.79
**History of psychiatric problems (*****N*** **=** **2,879)**		
No	2,766	96.08
Past only	28	0.97
Present only	63	2.19
Past and present	22	0.76
**Days between latest violence and first consultation (*****N*** **=** **2,505)**		
0–2	804	32.1
3–14	427	17.05
15–90	415	16.57
91–365	411	16.41
<365	448	17.88
**Type of perpetrators (*****N*** **=** **4,658)**		
Partner	1,197	25.5
Family member	558	11.88
Known civilian (non-family)	757	16.12
Unknow civilian	403	8.58
Military/Armed group	1,679	35.76
Other	64	1.36
**Number of perpetrators (*****N*** **=** **4,508)**		
One	2,596	57.59
Several	1,912	42.41

The main types of violence highlighted by the patient during the first session were quite diverse in nature, spanning from physical violence (52%) through witnessing physical violence (15%), rape (23%), insults/threats (12%), killing of a family member (8%), torture (7%), disappearance of a family member (5%) and other types of violence. Factors of vulnerability mentioned by the patient during the first session included destroyed property and/or loss of income (29%), lack of social support (21%), having been forced to flee (11%), having lost a loved one <2 years ago (10%) or more than 2 years ago (9%), being a female head of household (9%), experiencing marginalization (8%), among others. Less than five percent reported past and/or present psychiatric difficulties.

Most of the violent events had taken place in the patients' homes (60%), followed by the road (15%) or on a battlefield (13%). The alleged perpetrator was most often a member of the military or an armed group (35%), the patient's partner (26%), an unknown civilian (16%) or a family member (12%). Aggression by a single perpetrator (58%) was only slightly more common than aggression by several perpetrators (42%).

### Factors Associated With High Distress Prior to Receiving MHPSS

**Figure 1:**
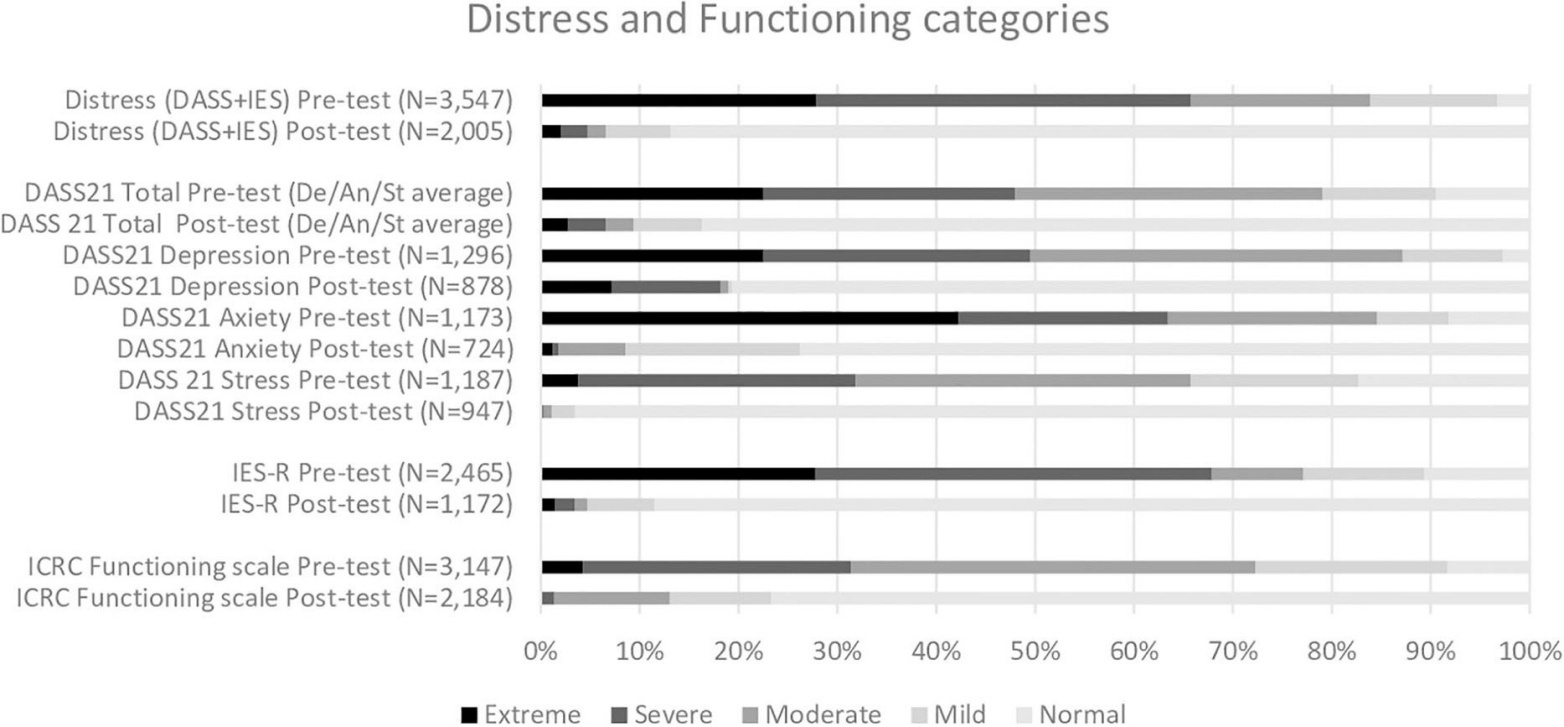
Pre and post severity categories of DASS21, IES-R and ICRC functionality scales.

The majority of the patients reported extreme or severe levels of distress at the time of enrolment (pre-test), regardless of whether the DASS21 or the IES-R scale was used (Figure 1). About a third of the patients also reported extreme or severe difficulties in daily functioning at the time of enrolment. See Appendix for full details on distress and functioning scores (Appendix I) and categories (Appendix II).

Several factors were associated with high levels of distress (cut-off defined by the median) at the time of enrollment (Table 4). Higher age was associated with increased odds of reporting high distress scores, with a peak between 45–55 years of age (aOR 3.60, *p* ≤ 0.0001). Medium education level was associated with increased odds (aOR 1.93, *p* ≤ 0.0001), as was occupations such as farming (aOR 2.85, *p* ≤ 0.0001) and other jobs (aOR 3.10, *p* < 0.001). Internally displaced patients had higher odds (aOR 2.63, *p* = 0.001) than residents, and both rape (aOR 2.23 *p* ≤ 0.0001) and caretaker neglect (aOR 2.14 *p* = 0.003) were also significantly associated with increased likelihood of presenting high distress scores at enrollment. Compared to patients who initiated MHPSS within 48 h following exposure to violence, patients who arrived between 91 and 365 days after were less likely to present high distress scores (aOR 0.52, *p* ≤ 0.0001) as were patients who arrived more than a year later (aOR 0.55, *p* ≤ 0.0001). The referral pathway mattered in that patients referred following a sensitization session in the community were more likely than self-referred patients to present high distress scores (aOR 1.47, *p* = 0.031), whereas patients referred following an information session inside the health facility were less likely (aOR 0.35, *p* = 0.013).

**Table 4:** Factors associated with high distress at baseline (*p*-value from Wald test, aOR adjusted by the other variables in the table and country).

**Variables**	**cOR (95%CI)**	***p*-value**	**aOR (95%CI)**	***p*-value**
**Age (*****N*** **=** **3,009)**				
2–17	Ref	–	Ref	–
18–24	1.50 (1.15; 1.96)	0.002	1.65 (1.05; 2.61)	0.031
25–34	1.77 (1.38; 2.27)	<0.0001	2.21 (1.38; 3.54)	0.001
35–44	1.78 (1.35; 2.339)	<0.0001	2.73 (1.64; 4.55)	<0.0001
45–54	2.03 (1.44; 2.86)	<0.0001	3.60 (1.98; 6.55)	<0.0001
55–81	1.40 (0.96; 2.04)	0.078	2.45 (1.21; 4.59)	0.005
**Education level (*****N*** **=** **3,297)**				
Illiterate	Ref	–	Ref	–
Primary	1.43 (1.23; 1.66)	<0.0001	1.18 (0.92; 1.51)	0.186
Secondary	2.28 (1.85; 2.79)	<0.0001	1.93 (1.37; 2.72)	<0.0001
Higher	1.42 (0.81; 2.50)	0.226	0.87 (0.40; 1.91)	0.737
**Current occupation (*****N*** **=** **3,051)**				
Unemployed	Ref	–	Ref	–
Student	0.61 (0.46; 0.80)	<0.0001	1.46 (0.86; 2.49)	0.166
Farming	1.15 (0.97; 1.36)	0.097	2.85 (2.17; 3.74)	<0.0001
Other jobs	1.02 (0.83; 1.25)	0.075	3.10 (2.09; 4.59)	<0.0001
**Status (*****N*** **=** **3,311)**				
Resident	Ref	–	Ref	–
Internally displaced	0.57 (0.46; 0.69)	<0.0001	2.63 (1.47; 4.72)	0.001
**Rape (*****N*** **=** **2,940)**				
No	Ref	–	Ref	–
Yes	1.35 (1.11; 1.59)	<0.0001	2.23 (1.65; 3.02)	<0.0001
**Caretaker neglect (*****N*** **=** **2,731)**				
No	Ref	–	Ref	–
Yes	1.38 (0.99; 1.91)	0.054	2.14 (1.29; 3.54)	0.003
**Days between latest violence and first consultation (*****N*** **=** **2,505)**				
0–2	Ref	–	Ref	–
3–14	1.08 (0.84; 1.3)	0.056	0.81 (0.57; 1.15)	0.244
16–90	1.19 (0.93; 1.54)	0.166	0.83 (0.60; 1.15)	0.244
91–365	0.69 (0.53; 0.88)	0.004	0.52 (0.38; 0.73)	<0.0001
<365	0.63 (0.49; 0.82)	<0.0001	0.55 (0.40; 0.77)	<0.0001
**Referred by (3,314)**				
Self	Ref	–	Ref	–
Sensitization session (in the community)	0.48 (0.25; 0.90)	0.022	1.47 (1.04; 2.08)	0.031
Information session (in the health facility)	0.48 (0.33; 0.69)	<0.0001	0.35 (0.15; 0.80)	0.013

Looking specifically at the IES-R (Table 5), factors associated with high scores at the time of enrollment were similar to those of overall distress when it came to age peaking between 45 and 54 years of age (aOR 5.88 *p* ≤ 0.0001), education peaking at secondary level (aOR 1.55, *p* ≤ 0.0001) and caretaker neglect (aOR 2.82, *p* = 0.001). In addition, having a missing relative stood out as a strong predictor of high IES-R pre-scores (aoR 4.95, *p* = 0.001) along with destroyed or lost property or income (aOR 2.15, *p* ≤ 0.0001). A linear trend was observed with regard to number of days since exposure to violence whereby more recent exposure was associated with higher odds of presenting high IES-R pre-scores. As for the referral pathway, high IES-R pre-scores were negatively associated with both referral from sensitization sessions (aOR 0.29, *p* = 0.008) and from the health facility itself (aOR 0.50, *p* = 0.005).

**Table 5:** Factors associated with high scores on IES-R at baseline (*p*-value from Wald test, aOR adjusted by the other variables in the table and country).

**Variables**	**cOR (95%CI)**	***p*-value**	**aOR (95%CI)**	***p*-value**
**Age (*****N*** **=** **2,425)**				
2–17	Ref	–	Ref	–
18–24	2.10 (1.55; 2.84)	<0.0001	2.85 (1.76; 4.63)	<0.0001
25–34	2.28 (1.71; 3.04)	<0.0001	3.20 (1.99; 5.14)	<0.0001
35–44	2.22 (1.62; 3.05)	<0.0001	3.97 (2.37; 6.66)	<0.0001
45–54	2.47 (1.67; 3.05)	<0.0001	5.88 (3.14; 11.01)	<0.0001
55–81	2 (0.31; 3.05)	0.002	3.98 (2.06; 7.71)	<0.0001
**Education (*****N*** **=** **2,452)**				
Illiterate	Ref	–	Ref	–
Primary	1.21 (1.01; 1.44)	0.03	1.36 (1.04; 1.77)	0.025
Secondary	1.86 (1.44; 2.39)	<0.0001	1.55 (1.06; 2.25)	0.023
Higher	1.22 (0.66; 2.25)	0.524	1.12 (0.51; 2.47)	0.77
**Other vulnerability factors (Ref** **=** **not mentioned by the patient)**				
Destroyed/lost property or income (*N* = 1,173)	1.03 (0.83; 1.30)	0.773	2.15 (1.54; 3.01)	<0.0001
Missing a relative (*N* = 1,868)	2.43 (1.25; 4.72)	0.009	4.95 (1.96; 12.49)	0.001
Caretaker neglect (for minors only) (*N* = 1,870)	2.13 (1.44; 3.16)	<0.0001	2.82 (1.672; 4.74)	<0.0001
**Number of days between exposure to latest violence and first consultation (*****N*** **=** **1,810)**				
0–2	Ref	–	Ref	–
3–14	1.01 (0.76; 1.34)	0.937	0.68 (0.46; 1.01)	0.053
15–90	1.17 (0.87; 1.55)	0.290	0.66 (0.46; 0.95)	0.023
91–365	0.68 (0.51; 0.91)	0.011	0.36 (0.25; 0.52)	<0.0001
365+	0.78 (0.59; 1.02)	0.067	0.34 (0.24; 0.48)	<0.0001
**Referred by (*****N*** **=** **2,455)**				
Self	Ref	–	Ref	–
Information sessions (in the health facility)	0.33 (0.16; 0.67)	0.002	0.29 (0.12; 0.76)	0.008
Local health structure	0.78 (0.55; 1.11)	0.166	0.50 (0.31; 0.81)	0.005

With regards to the likelihood of reporting high DASS21 scores at the time of enrollment (Table 6), a positive association was found between high depression scores and secondary education level (aOR 2.41, *p* = 0.003). A negative association was found with a duration of 91–365 days between exposure to latest violence and first MHPSS session (aOR 0.51, *p* = 0.027).

**Table 6:** Factors associated with high scores on the DASS21 subscales at baseline (*p*-value from Wald test, aOR adjusted by the other variables in the table and country).

	**cOR (95%CI)**	***p*-value**	**aOR (95%CI)**	***p*-value**
**DEPRESSION**				
**Education (1,270)**				
Illiterate	Ref	–	Ref	–
Primary	2.04 (1.56; 2.67)	<0.0001	1.33 (0.79; 2.25)	0.283
Secondary	3.44 (2.53; 4.69)	<0.0001	2.41 (1.34; 4.34)	0.003
Higher	1.79 (0.72; 4.48)	0.211	1	
**Number of days between exposure to latest violence and first consultation (*****N*** **=** **482)**				
0–2	Ref	–	Ref	–
3–14	0.93 (0.51; 1.69)	0.811	1.00 (0.54; 1.86)	0.983
15–90	1.04 (0.56; 1.94)	0.893	1.12 (0.59; 2.12)	0.723
91–365	0.52 (0.29; 0.93)	0.028	0.51 (0.28; 0.93)	0.027
<365	0.84 (0.33; 2.15)	0.716	0.88 (0.34; 2.30)	0.791
**ANXIETY**				
**Civil status (*****N*** **=** **1,173)**				
Single	Ref	–	Ref	–
Divorced/Separated	0.52 (0.29; 0.95)	0.032	7.22 (1.06; 48.96)	0.043
**Rape (*****N*** **=** **1,173)**				
No	Ref	–	Ref	–
Yes	7.18 (4.89; 10.54)	<0.0001	4.43 (2.44; 8.03)	<0.0001
**Other vulnerability factors (*****N*** **=** **1,173) (Ref** **=** **Not experienced)**				
Destroyed or lost property or income	1.45 (1.14; 1.84)	0.002	1.87 (1.11; 3.16)	0.019
Lack of social support/network	0.57 (0.41; 0.81)	0.001	2.58 (1.42; 4.71)	0.002
**Perpetrator (*****N*** **=** **739)**				
Partner	Ref	–	Ref	–
Family member	0.95 (0.52; 1.75)	0.877	0.45 (0.17; 1.20)	0.111
Known non-family member	3.62 (1.81; 7.24)	<0.0001	2.56 (0.94; 6.98)	0.067
Unknown civilian	4.96 (2.52; 9.74)	<0.0001	6.33 (2.30; 17.40)	<0.0001
Military/Armed group	1.70 (1.06; 2.71)	0.027	3.31 (1.56; 7.04)	0.002
Other/Not disclosed	1.89 (0.60; 5.91)	0.275	8.40 (1.51; 46.68)	0.015
**Number of days between exposure to violence and first consultation (*****N*** **=** **482)**				
0–2	Ref	–	Ref	–
3–14	0.53 (0.31; 0.91)	0.022	0.46 (0.23; 0.91)	0.025
15–90	0.50 (0.28; 0.87)	0.014	0.54 (0.27; 1.08)	0.08
91–365	0.44 (0.25; 0.79)	0.005	0.56 (0.27; 1.14)	0.108
<365	0.09 (0.04; 0.23)	<0.0001	0.18 (0.06; 0.55)	0.003
**STRESS**				
**Civil status (*****N*** **=** **1,164)**				
Single (incl. children)	Ref	–	Ref	–
Married	0.84 (0.63; 1.12)	0.237	2.32 (0.86; 6.25)	0.096
Divorced/Separated	0.32 (0.17; 0.59)	<0.0001	4.61 (0.05; 20.35)	0.043
Widow/er	0.41 (0.26; 0.65)	<0.0001	3.75 (0.85; 16.51)	0.081
**Status (*****N*** **=** **1,177)**				
Resident	Ref	–	Ref	–
Internally displaced	0.34 (0.26; 0.46)	<0.0001	0.43 (0.01; 0.26)	0.001
**History of psychiatric problems (*****N*** **=** **286)**				
None	Ref	–	Ref	–
Present	4.96 (1.77; 13.94)	0.002	5.93 (1.24; 28.43)	0.026

High DASS21 anxiety at enrollment odds were more than seven times more likely in patients who were divorced or separated (aOR 7.22, *p* = 0.043) than single patients, and more than four times higher in patients who had experienced rape (aOR 4.43, *p* ≤ 0.001) compared to those who did not report having experienced this type of violence. The types of perpetrators most strongly associated with high DASS21 anxiety at enrollment were unknown civilians (aOR 6.33, *p* ≤ 0.0001), military or armed group (aOR 3.31, *p* = 0.002) and other/not disclosed perpetrators (aOR 8.40, *p* = 0.015). Finally, compared to patients consulting within 48 h following exposure to violence, patients consulting 3–14 days after were less than half as likely to present high DASS21 anxiety symptoms (aOR 0.46, *p* = 0.025) and patients consulting more than 1 year after were five times less likely (aOR 0.18, *p* = 0.003) to do so.

The strongest predictor of high DASS21 *stress* odds at enrollment was the presence of *psychiatric problems*, which made patients almost six times more likely. Patients reporting high levels of stress at the time of enrollment were also characterized by a *higher level of education*, with patients having finished secondary school being five times more likely than illiterate patients to report high stress scores at the time of enrollment. *Married* patients were five times more likely than singles to show high levels of stress at the time of enrollment, even when controlling for other factors such as age and the type of perpetrator, i.e., the patient's partner, which would indicate domestic violence. Internally displaced people appeared three times less likely than residents to report high levels of stress at the time of enrollment.

Factors associated with low functioning prior to MHPSS support (Table 7) were being divorced or separated (aOR 2.09, *p* ≤ 0.0001), having experienced rape (aOR 1.36, *p* = 0.015), having experienced forced marriage (aOR 3.59, *p* = 0.017) and having lost a loved one more than 2 years ago due to natural causes (aOR 2.33, *p* ≤ 0.0001). Compared to violence committed by the partner (i.e., domestic violence), odds were higher if the perpetrator was a member of the military or an armed group (aOR 1.60, *p* = 0.010), another family member (aOR 1.47, *p* = 0.023) and particularly if the patient did not wish to disclose the type of perpetrator (aOR 6.12, *p* = 0.028).

**Table 7:** Factors associated with low functioning at baseline (*p-value from Wald test, aOR adjusted by the other variables in the table and country).

**Variables**	**cOR (95%CI)**	*p*-value*	**aOR (95%CI)**	*p*-value*
**Civil status (*****N*** **=** **5,407)**				
Single (incl. children)	Ref	–		
Divorced/Separated	0.94 (0.74; 1.20)	0.624	2.09 (1.49; 2.93)	<0.0001
**Main types of violence highlighted by the patient during the first consultation (Ref** **=** **Not Experienced)**				
Rape (*N* = 2,767)	1.62 (1.37; 1.90)	<0.0001	1.36 (1.06; 1.75)	0.015
Forced marriage (*N* = 2,559)	2.97 (1.36; 6.47)	0.006	3.59 (1.26; 10.25)	0.017
**Natural death of loved one more than 2 years ago (2,598)**				
No	Ref	–	Ref	–
Yes	1.29 (0.99; 1.66)	0.05	2.33 (1.70; 3.19)	<0.0001
**Type of perpetrator(s) (*****N*** **=** **2,903)**				
Partner	Ref	–	Ref	–
Family member	0.60 (0.45; 0.78)	<0.0001	1.47 (1.06; 2.06)	0.023
Known civilian (non-family)	0.71 (0.56; 0.89)	0.004	1.17 (0.85; 1.61)	0.34
Unknow civilian	0.62 (0.46; 0.84)	0.002	1.39 (0.95; 2.04)	0.094
Military/Armed group	0.24 (0.20; 0.300)	<0.0001	1.60 (1.12; 2.29)	0.01
Other/Not disclosed	1.08 (0.57; 2.05)	0.82	6.12 (1.22; 30.80)	0.028

### Factors Associated With Improvement Following MHPSS

The factors associated with improvement following MHPSS (Table 8) varied greatly depending on the domain. When looking at improvement of psychological distress (Table 9), the most important factor was having high distress at baseline (aOR 28.70, *p* ≤ 0.0001), i.e., having room for improvement. A clear linear trend was seen between likelihood of improved distress and number of sessions attended, with patients attending seven or more sessions being almost seven times more likely to improve than patients who received only pre- and post-assessments (aOR 6.75, *p* = 0.001). Both students and farmers were only half as likely to improve as unemployed patients.

**Table 8:** Characteristics of the MHPSS.

**Variable**	***n***	**%**
**Days between latest violence and first consultation (*****N*** **=** **2,505)**		
0–2	804	32.1
3–14	427	17.05
15–90	415	16.57
91–365	411	16.41
<365	447	17.88
**Number of individual sessions excluding pre- and post-assessment sessions (*****N*** **=** **3,694)**		
0	647	17.51
1–2	1,685	45.61
3–4	1,319	35.71
5–10	43	1.16
**Number of group sessions excluding pre- and post-assessment sessions (*****N*** **=** **42)**		
1–2	24	16.11
3 or more	18	12.06
**Referrals (*****N*** **=** **2,505)**		
Other psychological (specialist)	6	0.13
Other psychosocial	8	0.17
Health	494	10.64
Economic/Livelihood	8	0.17
ICRC Protection	65	1.4
Legal support	29	0.62
Education/School	36	0.78

**Table 9:** Factors associated with improved distress following MHPSS (*p-value from Wald test, aOR adjusted by the other variables in the table and country).

**Variable**	**Crude OR (95%CI)**	*p*-value*	**aOR (95%CI)**	*p*-value*
**Current occupation (*****N*** **=** **3,051)**				
Unemployed	Ref	–	Ref	–
Student	0.47 (0.32; 0.70)	<0.0001	0.47 (0.24; 0.91)	0.025
Farming	0.98 (0.76; 1.27)	0.906	0.48 (0.29; 0.78)	0.003
Other jobs	0.73 (0.05; 11.82)	0.827	1	
**High distress at baseline (*****N*** **=** **1,769)**				
No	Ref	–	Ref	–
Yes	28.03 (21.72; 36.17)	<0.0001	28.70 (20.92; 39.37)	<0.0001
**Number of individual sessions (excluding enrollment and closure sessions) (*****N*** **=** **1,529)**				
0	Ref	–	Ref	–
1–3	2.20 (1.43; 3.40)	<0.0001	2.09 (1.19; 3.69)	0.011
4–6	1.68 (1.10; 2.56)	0.016	3.49 (1.75; 6.94)	<0.0001
7 or more	2.39 (1.08; 5.29)	0.031	6.75 (2.13; 21.34)	0.001

Looking at improvement on the IES-R scale (Table 10), associated factors included having high IES-R scores at baseline (aOR 33.70, *p* ≤ 0.0001), a mother heading a household (aOR 9.23, *p* ≤ 0.0001), having low functioning at baseline (aOR 3.23, *p* ≤ 0.0001) and lacking social support (aOR 2.77, *p* = 0.007).

**Table 10:** Factors associated with improved IES-R total score (38 points or more) following MHPSS (*p-value from Wald test, aOR adjusted by the other variables in the table and country).

**Variable**	**Crude OR (95%CI)**	*p*-value*	**aOR (95%CI)**	*p*-value*
**High IES-R scores at baseline (*****N*** **=** **1,094)**				
No	Ref	–	Ref	–
Yes	27.85 (20.12; 38.55)	<0.0001	33.70 (17.16; 66.16)	<0.0001
**Low functioning at baseline (*****N*** **=** **1,003)**				
No	Ref	–	Ref	–
Yes	1.98 (1.54; 2.56)	<0.0001	3.23 (1.88; 5.55)	<0.0001
**Other vulnerability factors**				
Mother head of household **(*****N*** **=** **672)**	17.69 (7.61; 41.10)	<0.0001	9.23 (3.22; 26.41)	<0.0001
Lack of social support/Network **(*****N*** **=** **673)**	1.69 (1.07; 2.66)	0.024	2.77 (1.33; 5.80)	0.007

The main factor associated with improvement on the DASS21 (Table 11) depression subscale was high DASS21 depression scores at baseline (aOR 29.93, *p* ≤ 0.0001). A negative association was found with patients having suffered destruction or loss of property or income (aOR 0.58, *p* = 0.005).

**Table 11:** Factors associated with improvement on DASS21depression scores (14 points or more), anxiety scores (13 points or more) and stress scores (16 points or more) following MHPSS (*p-value from Wald test, aOR adjusted by the other variables in the table and country).

**Variable**	**Crude OR (95%CI)**	*p*-value*	**aOR (95%CI)**	*p*-value*
**DEPRESSION**				
**High DASS depression scores at baseline (*****N*** **=** **1,134)**				
No	Ref	–	Ref	–
Yes	22.59 (16.37; 31.16)	<0.0001	29.93 (18.70; 47.91)	<0.0001
**Destroyed or lost property or income (*****N*** **=** **876)**				
No	Ref	–	Ref	–
Yes	0.47 (0.36; 0.62)	<0.0001	0.58 (0.40; 0.85)	0.005
**ANXIETY**				
**High DASS21 anxiety scores at baseline (*****N*** **=** **1,111)**				
No	Ref	–	Ref	–
Yes	36.64 (25.83; 51.98)	<0.0001	44.56 (24.28; 81.78)	<0.0001
**Number of days between exposure to latest violence and first consultation (*****N*** **=** **429)**				
0–2	Ref	–	Ref	–
3–14	0.62 (0.35; 1.08)	0.093	0.82 (0.37; 1.82)	0.631
15–90	0.81 (0.46; 1.41)	0.449	1.25 (0.56; 2.81)	0.592
91–365	0.59 (0.33; 1.05)	0.073	0.72 (0.32; 1.63)	0.435
<365	0.10 (0.04; 0.29)	<0.0001	0.23 (0.06; 0.93)	0.039
**STRESS**				
**High DASS21 stress scores at baseline (*****N*** **=** **937)**				
No	Ref	–	Ref	–
Yes	23.62 (16.74; 33.34)	<0.0001	19.88 (13.78; 28.68)	<0.0001
**Destroyed/Lost property/income (*****N*** **=** **937)**				
Not reported	Ref	–	Ref	–
Reported	0.51 (0.39; 0.67)	<0.0001	0.60 (0.42; 0.87)	0.006

Having high DASS21 anxiety scores at baseline was by far the strongest predictor of improvement on the DASS21 anxiety subscale. In addition, patients who consulted more than a year after exposure to violence had lower odds than patients consulting within 48 h (aOR 0.23, *p* = 0.039).

When it came to improved DASS21 stress scores, patients with high scores at baseline (aOR 19.88, *p* ≤ 0.0001) and patients from DRC (aOR 1.83, *p* = 0.036) had the highest odds. Having suffered destruction or loss of property or income due to violence was associated with less likelihood of improving stress scores following MHPSS (aOR 0.60, *p* = 0.006).

Factors associated with improved functioning (Table 12) included low functioning at enrollment (aOR 27.53, *p* ≤ 0.0001) and low distress at enrollment (aOR 3.90, *p* ≤ 0.0001). A clear link was observed between improved functioning and number of individual sessions, whether four to six sessions (aOR 3.12, *p* = 0.003) or seven or more sessions (aOR 11.61, *p* = 0.050). Having been a victim of trafficking was associated with less improvement (aOR 0.13, *p* = 0.049) as was having experienced discrimination (aOR 0.48, *p* = 0.032) and having been forced to flee (aOR 0.16, *p* = 0.003).

**Table 12:** Factors associated with improved functioning (<5 points) following MHPSS (*p-value from Wald test, aOR adjusted by the other variables in the table and country).

**Variables**	**cOR (95%CI)**	*p*-value*	**aOR (95%CI)**	*p*-value*
**Trafficking (*****N*** **=** **1,724)**				
No	Ref	–	Ref	–
Yes	0.68 (0.16; 2.87)	0.604	0.13 (0.02; 0.99)	0.049
**Discrimination (*****N*** **=** **1,725)**				
No	Ref	–	Ref	–
Yes	1.07 (0.76; 1.51)	0.709	0.48 (0.24; 0.94)	0.032
**Forced to flee (*****N*** **=** **1,724)**				
No	Ref	–	Ref	–
Yes	0.04 (0.02; 0.09)	<0.0001	0.16 (0.05; 0.53)	0.003
**Low functioning at baseline (binary) (*****N*** **=** **2,147)**				
No	Ref	–	Ref	–
Yes	7.36 (6.08; 8.90)	<0.0001	27.53 (18.26; 41.51)	<0.0001
**High distress at baseline (binary) (*****N*** **=** **1,630)**				
No	Ref	–	Ref	–
Yes	2.45 (2.00; 3.00)	<0.0001	3.90 (2.62; 5.79)	<0.0001
**Number of individual sessions (excluding enrollment and closure sessions) (*****N*** **=** **2,015)**				
0	Ref	–	Ref	–
1–3	1.49 (0.93; 2.38)	0.1	1.74 (0.92; 3.31)	0.127
4–6	3.95 (2.46; 6.34)	<0.0001	3.12 (1.48; 6.55)	0.003
7 or more	4.89 (2.04; 11.72)	<0.0001	11.61 (1.00; 135.30)	0.05

## Discussion

In this section we will first discuss the recurrent characteristics of the *patient*, including the exposure to violence, that predicted high distress and low functioning at baseline and/or improvement following the intervention (Table 13). Second, we will discuss characteristics of the *intervention* linked to improvement. Lastly, we will present a series of programme recommendations aiming at further tailoring the MHPSS intervention to the needs of victims of violence supported at primary healthcare level.

**Table 13:** Summary of factors associated with distress and functioning scores before and after MHPSS.

	**Increased odds**	**Reduced odds**
**Factors associated with high distress and low functioning prior to MHPSS**		
**Distress(IES-R** **+** **DASS21)**	Age peaking at 45-54, Secondary level of education, Farming or other jobs, IDP, Rape, Caretaker neglect, Referred from sensitization session	<3 Months since violence, Referred from information session
**IES-R**	Age peaking at 45-54, Primary and secondary levels of education, Missing a relative, Caretaker neglect, Destroyed or lost property or income	Referral from information session or health structure, Number of days since violence (linear trend)
**DASS21 Depression**	Secondary education	91–365 days since violence
**DASS21 Anxiety**	Divorced or separated, Rape, Perpetrator: Unknown civilian, military or armed group, other/not disclosed	Days since exposure to violence: 3-14 days and <365 days
**DASS21 Stress**	Divorced or separated,	IDP
	Present psychiatric problem	
**Functioning**	Divorced or separated, Natural death of a loved one more than 2 years ago, Perpetrator: Military or armed group, or other/not disclosed.	
	**Increased odds**	**Reduced odds**
**Factors associated with improvement following MHPSS**		
**Distress (IES-R** **+** **DASS21)**	Number of individual sessions (linear trend), High distress scores at baseline	Students, Farmers
**IES-R**	High IES-R at baseline, Low functioning at baseline, Mother head of household, Lack of social support	
**DASS21 Depression**	High depression scores at baseline	Destroyed/lost property or income
**DASS21 Anxiety**	High anxiety scores at baseline	<365 days since violence
**DASS21 Stress**	High stress scores at baseline	Destroyed/lost property or income
**Functioning**	Low functioning at baseline, High distress at baseline, <3 Individual sessions	Trafficking, Discrimination, Forced to flee

### Characteristics of the Patients

#### Age

The finding that patients aged between 45 and 54 are the most likely to present high distress prior to receiving MHPSS matches the finding of the recent Cochrane study (1) insofar as a decline in distress levels was seen after 55 years of age. However, the peak in PTSD symptoms at 25 years of age was not found in this study. The fact that age was not associated with treatment outcome suggests that the MHPSS intervention addresses the needs of patients of all ages.

#### Caretaker Neglect

Minors experiencing caretaker neglect were significantly more likely than average to report high distress at baseline. Indeed, caretaker neglect should remain a red flag that calls for the counselor's immediate attention. The fact that this neglect did not correlate with lesser improvement indicates that the intervention by and large addresses the needs of the children. However, more research is needed to better understand to what extent the intervention in its current form is adapted to the needs of children in general and those experiencing caretaker neglect in particular.

#### Divorce

Both high stress and low functioning at baseline correlated with being divorced or separated. This would indicate that—independently of exposure to violence—being divorced or separated aggravates the patient's stress and functioning levels. The fact that divorced or separated patients improved as much as average suggests that the MHPSS adequately addressed their needs.

#### Destroyed or Lost Property or Income

This was a predictor of higher PTSD symptoms at baseline as well as less improvement in depression and stress following the intervention. This finding is consistent with previous studies that found a link between financial means and mental health (15) and suggests that while the counselor may be able to address some of the psychological consequences of destroyed or lost property or income, symptoms of depression and stress remain high if the financial consequences of the losses are not addressed.

#### Education Level

Secondary education was associated with higher distress in general at enrolment, particularly PTSD and depression. This finding is inconsistent with other studies that have looked at the link between educational level and psychological distress in Uganda (16) and Africa (17). We may also speculate that since well-educated people have found to be more involved in politics (18), they more easily become political targets of violence (political affiliation was not monitored) or that better educated are more easily startled by violence. On the other hand, it may also be that the somewhat complex vocabulary used in the distress scales was more easily understood by the well-educated patients. This would also explain why there was no link between education level and the functioning scale that uses more simple language as well as visual illustrations. Either way, the fact that education level did not correlate with treatment outcome indicates that the intervention is adapted to patients of all educational levels.

#### Missing Relative

Missing a relative stood out as a factor significantly associated with high levels of distress prior to receiving MHPSS. This link has been established in post-conflict settings (19), however, the finding that it also plays a significant role in determining levels of distress in contexts of ongoing violence was somewhat unexpected. The fact that missing a relative was not significantly linked to the outcome suggests that the MHPSS in its current form adequately addresses the needs of this sub-group of patients.

#### Occupation

Occupation was not significantly associated with levels of psychological distress and functioning prior to MHPSS. However, following MHPSS, psychological distress among students (11% of the study population) and farmers (38%) improved significantly less than among patients with other jobs (23%) and those unemployed (28%).

#### Perpetrator Profile

Violence committed by members of the military or an armed group was associated with lower functioning at baseline, underlining the debilitating consequences that violence committed by these types of perpetrators can have on the daily functioning of their victims. The fact that perpetrator profile did not correlate with treatment outcome indicates that the intervention is equally suitable for victims of violence committed by civilians and weapon bearers.

#### Rape

Standing out as a particularly debilitating type of violence, rape correlated significantly with high psychological distress prior to MHPSS. This result is consistent with the findings of the recent MSF CAR study (2). The fact that patients who had experienced rape improved as much as average indicates that the intervention in its current form addresses the psychological needs stemming from this type of violence.

#### Referral Pathway

The fact that significantly lower level of distress was reported by patients referred after an ICRC information session or by health personnel indicates a need for more clarity when it comes to explaining psychological distress and identifying patients in need of MHPSS.

### Characteristics of the Intervention

#### Number of Individual Sessions

The number of individual sessions correlated with improvement in both distress (lower) and functioning (higher). While some therapeutic approaches such as single-session therapy (20) are designed for brief encounters, the short-term solution-oriented therapy offered to this cohort does require a series of sessions to be effective. The larger the number of sessions, the greater the likelihood of improvement. This finding indicates that despite some methodological limitations of the study, changes in levels of distress and functioning appear strongly linked to the MHPSS intervention.

#### Referrals

Referrals did not significantly correlate with treatment outcome. It would appear that patients who have suffered important economic losses as a result of conflict would have benefited from a referral to an economical services provider. ICRC economic security projects are usually offered after the MHPSS has ended. Psychological outcomes of such assistance are not systematically monitored, including for the cohort involved in this study.

### Programme Recommendations

1. Triage: Health personnel and awareness raisers carrying out information sessions may benefit from more training on identifying patients with specific MHPSS needs to better filter the patients that they refer to the counselor. At the counselor's level, it would seem relevant to increase the filtering of patients based on the distress and functioning scales in order to identify and focus on the most vulnerable.
2. Increase number of sessions per patient: We saw a clear link between number of individual sessions and improved distress and functioning. Four sessions—excluding enrolment and closure—should be the target taken into consideration when budgeting, for example, the reimbursement of transportation costs.
3. Address depression: The current approach is well-tailored to PTSD and anxiety in general. However, for almost 20% of the patients, depression scores remain severe or extremely severe following MHPSS. There appears to be a need to increase training of counselors on therapeutic approaches with regard to depression and the effect of more long-term reactions to witnessing and/or experiencing multiple violent events.
4. Financial assistance: We found a significantly smaller improvement in anxiety and stress symptoms among patients who experienced destruction or loss of property or income. This would indicate that MHPSS alone is not sufficient and that this group of patients need further support in facing the economic consequences of violence. A more systematic inclusion of a financial component of projects for victims of violence is recommended, along with regular monitoring of MHPSS outcomes of financial support.
5. Explore the role of education: a qualitative study can be considered to better understand the tendency of patients with primary and secondary education levels to report higher levels of psychological distress prior to receiving MHPSS.

As the very first study of victims of violence benefiting from ICRC MHPSS integrated into health-care facilities across Africa, the main attributes of this study constitute the large sample size and real-life setting. However, the study also has some limitations to take into consideration. First, as there was no control group we cannot state with certainty that changes observed were in fact due to the MHPSS received. Second, the nature of the conflict setting made it very difficult for patients to access the health facilities for MHPSS follow-up sessions and 43% of the patients did not have a post-assessment. Also, in these acute circumstances clinical care was prioritized over systematic data collection, leading to missing values for different variables. Third, the use of self-reported scales could have created an information bias, however, while some patients may overstate and other patients may understate, any change in symptoms would still be measured reliably. Finally, the fact that we do not know the distress and functioning levels of the patients before exposure to violence nor in the long term following the MHPSS intervention makes it difficult to state with certainty what impact the violence had on the patients and to what extent the MHPSS was useful in the long term.

Despite these limitations, the findings of this study underline that exposure to violence in the context of armed conflict is associated with high levels of psychological distress and low functioning. Fortunately, strong associations between MHPSS intervention, reduced distress and increased daily functioning underline that this type of support adequately addresses the psychological needs of victims of violence. To further address these needs, it should be prioritized to intervene quickly, increase the number of individual sessions per patient, address symptoms of depression and tackle financial needs deriving from exposure to violence.

## Data Availability Statement

The raw data supporting the conclusions of this article will be made available by the authors, without undue reservation.

## Author Contributions

IA and MY: literature search and collected the data. IA and RR: conceived and designed the analysis. IA: performed the analysis and wrote the paper. MY, IH, and RR: commented. All authors contributed to the article and approved the submitted version.

## Conflict of Interest

The authors declare that the research was conducted in the absence of any commercial or financial relationships that could be construed as a potential conflict of interest.

## Abbreviations

CAR: Central African Republic
DASS21: Depression Anxiety and Stress Scale (21 items)
DRC: Democratic Republic of the Congo
IDP: Internally displaced persons
IES-R: Impact of Events Scale – Revised
ICRC: International Committee of the Red Cross
MHPSS: Mental health and psychosocial support
PHC: Primary health care.
