# Supplementary material for: Integrating Mental Health and Psychosocial Support Into Health Facilities in Conflict Settings: A Retrospective Review From Six African Countries
Source: Front Public Health. 2020 Dec 11;8:591369. doi: 10.3389/fpubh.2020.591369 (PMC7759644; doi:10.3389/fpubh.2020.591369)
Supplement: Supplementary file 1 [file Table_1.docx]

**Appendix**

|  | **Mean (SD)** | **95%CI mean** | ***P*-value** | **Range** | **Median** | **% of at least 1 gain** |
| --- | --- | --- | --- | --- | --- | --- |
| **Pre DASS (N=861)** | 61.05 (18.72) | 59.80; 62.30 | <0.0001 | 2-116 | 62 |  |
| **Post DASS (N=972)** | 17.12 (10.40) | 16.47; 17.78 | <0.0001 | 4-108 | 7.13 |  |
| **Difference in DASS (N=677)** | 41.39 (18.07) | 40.03; 42.76 | <0.0001 | - 16 - 96 | 40 | 671 (99.11%) |
| **Pre IES (N=2,465)** | 48.73 (19.71) | 47.95; 49.51 | <0.0001 | 1-88 | 48 |  |
| **Post IES (N=1,172)** | 11.36 (12.15) | 10.67; 11.06 | <0.0001 | 1-83 | 8 |  |
| **Difference in IES (N=1,094)** | 38.93 (20.37) | 37.7; 40.14 | <0.0001 | - 44 - 83 | -38 | 1,064 (97.26%) |
| **Pre Functioning (N=3,147)** | 7.02 (2.82) | 47.95; 49.51 | <0.0001 | 1-14 | 7 |  |
| **Post Functioning (N=2,184)** | 12.19 (2.40) | 12.09; 12.29 | <0.0001 | 1-21 | 13 |  |
| **Difference Functioning (2,147)** | 5.40 (3.12) | 5.27; 5.53 | <0.0001 | -1 - 12 | 6 | 2,009 (93.58%) |

***Appendix I.*** *Distress and Functioning Scores*

| **Category** | **Extreme n (%)** | **Severe n (%)** | **Moderate n (%)** | **Mild n (%)** | **Normal n (%)** |
| --- | --- | --- | --- | --- | --- |
| **DASS21 total score**  Pre-test (average)  Post-test (average)  Depression subscale  Pre-test (N=1,296)  Post-test (N=878)  Anxiety subscale  Pre-test (N=1,173)  Post-test (N=724)  Stress subscale  Pre-test (N=1,187)  Post-test (N=947) | (22.16)  (2.73)  292 (22.53)  60 (6.83)  471 (40.15)  9 (1.24)  45 (3.79)  1 (0.11) | (25.03)  (3.78)  349 (26.93)  93 (10.59)  236 (20.12)  4 (0.55)  333 (28.05)  2 (0.21) | (30.55)  (2.76)  489 (37.73)  6 (0.68)  294 (20.06)  49 (6.77)  402 (33.87)  8 (0.84) | (11.32)  (6.78)  131 (10.11)  3 (0.34)  81 (6.91)  128 (17.68)  201 (16.93)  22 (2.32) | (9.27)  (82.46)  35 (2.70)  667 (77.11)  91 (7.76)  534 (73.76)  206 (17.35)  914 (96.52) |
| **IES-R total score**  Pre-test (N=2,465)  Post-test (N=1,172) | 685 (27.79)  17 (1.45) | 989 (40.12)  23 (1.96) | 227 (9.21)  15 (1.28) | 301 (12.21)  80 (6.83) | 263 (10.67)  1,037 (88.48) |
| **Distress (DASS+IES)**  Pre-test (N=3,421)  Post-test (N=2,005) | 954 (27.89)  40 (2.00) | 1,295 (37.85)  56 (2.79) | 622 (18.18)  36 (1.80) | 439 (12.83)  131 (6.53) | 111 (3.25)  1,742 (86.88) |
| **ICRC functioning scale**  Pre-test (N=3,147)  Post-test (N=2,184) | 133 (4.23)  3 (0.14) | 854 (27.14)  27 (1.24) | 1,287 (40.90)  255 (11.68) | 613 (19.48)  223 (10.21) | 260 (8.26)  1,676 (76.74) |

***Appendix II:*** *Distress and Functioning categories*
